# Development and psychometric properties of Female Infertility Stigma Instrument (ISI-F): A sequential mixed method study

**DOI:** 10.1186/s12905-022-02139-5

**Published:** 2022-12-30

**Authors:** Mahboube Taebi, Nourossadat Kariman, Ali Montazeri, Hamid Alavi Majd, Maryam jahangirifar

**Affiliations:** 1 Reproductive Sciences and Sexual Health Research Center, Isfahan University of Medical Sciences, Isfahan, Iran; 2 Midwifery and Reproductive Health Research Center, Department of Midwifery and Reproductive Health, Shahid Beheshti University of Medical Sciences, P.O. Box 1996835119, Tehran, Iran; 3 Health Metrics Research Centre, Iranian Institute for Health Sciences Research, ACECR, Tehran, Iran; 4 Department of Biostatistics, School of Allied Medical Sciences, Shahid Beheshti University of Medical Sciences, Tehran, Iran; 5 Faculty of Medicine, Nursing and Health Sciences, School of Nursing and Midwifery, Monash University, Melbourne, Australia

**Keywords:** Infertility, Stigma, Factor analysis, Reliability and validity, Psychometric

## Abstract

**Background:**

Infertility stigma is a hidden burden that overshadows the dimensions of reproductive and sexual health in infertile women. The aim of this study was to develop and evaluate the psychometric properties of the Female Infertility Stigma Instrument (ISI-F).

**Methods:**

This mixed method study with sequential exploratory design was conducted in qualitative and quantitative phases. In the first phase, the initial item pool of the Female Infertility Stigma Instrument (ISI-F) was generated using in-depth interviews. In the quantitative phase, psychometric properties of the ISI-f including content, face and construct validity, as well as reliability (internal consistency and stability) were assessed. Exploratory factor analysis was performed on the collected data from 300 infertile women for evaluation of construct validity. Data was analyzed using SPSS version 20. This study has followed the Mixed Methods Article Reporting Standards checklist.

**Results:**

The final version of ISI-F had 20 items. Total CVI and CVR were 0.94 and 0.87, respectively. Explanatory factor analysis identified 3 main factors that explained 54.013% of the variance. These factors consisted of stigma profile (7 items), self-stigma (6 items) and escaping from stigma (7 items). Internal consistency and stability of the ISI-F has been approved by Cronbach’s alpha, McDonald's Omega (0.909, 0.916) and Intraclass Correlation Coefficient (ICC = 0.878).

**Conclusion:**

The Female Infertility Stigma Instrument (ISI-F) is a valid and reliable tool for evaluation of the perceived female infertility stigma, that was developed in this study.

## Background

Infertility is defined as inability to get pregnant after one year of unprotected sexual intercourse and has become a global problem. According to international statistics, about 186 million individuals are suffering from infertility around the world and it affects about 10 to 15 percent of people during their fertile age [1–3]. Infertility is considered as one of the most destructive crisis in the couples’ lives [4]. Due to realistic or unrealistic thoughts, infertile individuals usually feel unacceptance from the society and lack of empathy from others and therefore, they would feel isolated form the world of fertile people. Feeling isolated, social stigma and loss of control constitutes their identity [5, 6]. Although the rate of infertility is almost equal between men and women; most societies have held women responsible for infertility and this would cause infertile women feel guilty and threaten their self-esteem [7, 8].

Women have described infertility as the most saddening experience of their life [9, 10]. Motherhood is a part of identity for many women and they would appear more vunerable in the case of infertility in comparison to men and would expereince more stigma [2, 11]. Infertility threaten the security of women and they would burden a great amount of stress due to the stigma. For most women, infertility is a hidden stigma which is associated with the feelings of shame and secrecy [12, 13]. Stigma is defined as a negative feeling of being different from others in the society and being unlike the social norms. From Goffman 's point of view, stigma is a discrediting social difference that leads to a “spoiled identity"[14], and a theoretical overview of the stigma concept showed that stigma is in the form of "public stigma; self-stigma; stigma by association and structural stigma"; it is state that public stigma is the main root of the other forms of stigma [15]. If infertility would be experienced as a stigma, it would deprive the infertile individual from the potential support sources that would lead to feelings of anxiety, stress [16, 17], guilt, stigma [18] and disruption of relationships [3]. In our qualitative study, the infertile women perceive stigma profile as verbal, social and same sex stigma. They also experience self-stigma as negative feelings, and devaluation. Defensive mechanisms that women use were escaping from stigma, acceptance and hiding the infertility [12]. Stigma is associated with the mental and social dimensions of infertility and cause the infertile individual to be unable to accept themselves as someone like others in the society [11]. Considering the adverse effect of infertility on the mental status and relationships of the individuals [7, 19], well-designed tool with approvd reliability and validity are necessary to evaluate the percieved stigma of the infertility.

For evaluating the infertility stigma, some studies have used the general tools for evaluation of stigma just by adding the word “infertility” into these tools [16, 20–22]. Considering that morbidity and physical problems have various social, mental and psychological consequences, general questionnaires are not able to answer the specific issues that are raised by a specific condition such as infertility. For examples in neurological diseases, questionnaires are mostly designed based on social rejection and discriminatory behaviors [23], For AIDS, stigma questionnaire is mostly consisted of concerns about disease disclosure, having negative image of themselves and concerns about social behaviors [24]. Even for a disease such as type 2 diabetes, stigma questionnaire is about different behaviors, identity concerns and judgment [25]. For associative stigma of mental illness, the dimensions of violence-dangerousness, disability, and irresponsibility-lack of competence have been found [26]. It reveals that for each medical issue, based on their type and consequences, the type of stigma is different and general questionnaires could not address the specific issues caused by that condition. For example, in AIDS, people are mostly concerned about the transmission of the disease and stigma would reveal itself as social rejection and even violent behaviors but for a condition like infertility, this type of stigma might not be common. Even female and male infertility might be different regarding their experienced stigma [24]. Therefore, it is necessary to design a specific questionnaire for this purpose. The present study was conducted for designing and psychometric assessment of the female infertility stigma instrument.

## Material and methods

**Fig. 1:**
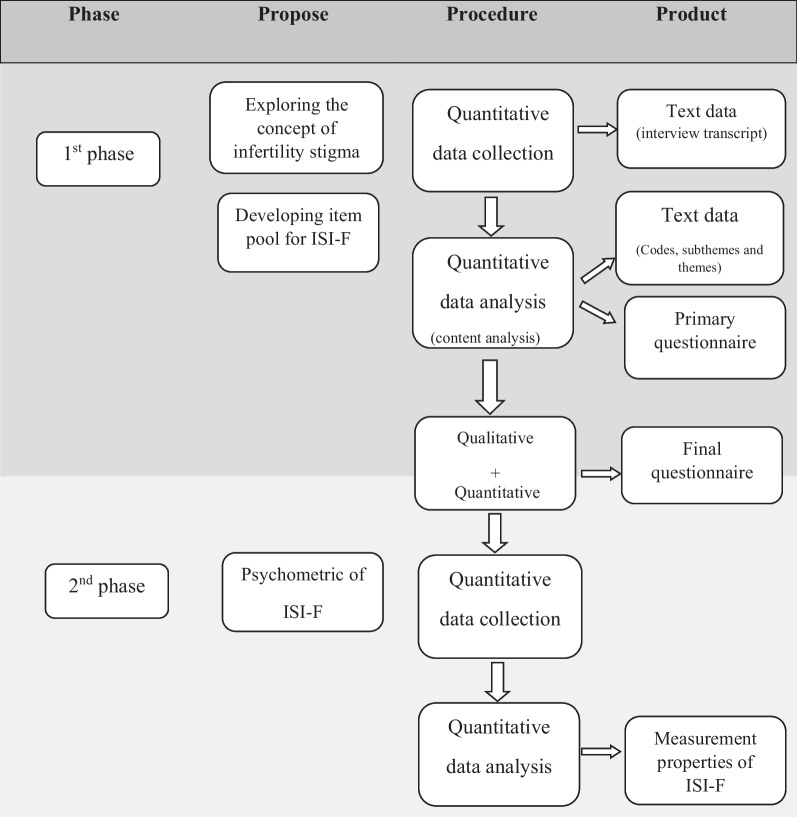
Flowchart of the sequential exploratory mixed method study [27]. ISI-F; Female Infertility Stigma Instrument

This exploratory, sequential mixed-method study was conducted with the qualitative-quantitative sequencing design. The protocol of this study has been published before. The diagram of the study has been presented in Fig. 1[27]. The method will be presented briefly here. The present article adheres to the EQUATOR guidelines for reporting research using the Mixed Methods Article Reporting Standards (MMARS) checklist.

### Qualitative phase

i. *Item generation* In this phase, the ISI-F (Female Infertility Stigma Instrument) items were generated using the data from interviews with infertile women who refer to the Isfahan fertility and infertility center, Isfahan, Iran from 2019 to 2020. The qualitative phase of the study has been published before [12] Using the data from the interviews, an initial pool of 108 items was generated. After a careful review by the research team, the number of items was reduced to 55. Upon initial agreement, the items were scored on a 5-point Likert scale from 1 (totally disagreed) to 5 (totally agreed). The questionnaire was prepared for evaluation of psychometric criteria.

### Quantitative phase

The psychometric indexes of ISI-F including content, face and construct validity, as well as reliability (internal consistency and stability) were measured in this phase.

i. *Content validity* Content validity of the instrument was carried out using qualitative and quantitative approaches.
  In the qualitative content validity method, the opinions of experts were evaluated. The purposive sampling was used to invite 10 experts who were well distinguished in the fields of qualitative research, instrument development, and health sciences. The faculty members of nursing, midwifery, epidemiology, psychology, psychiatry and reproductive health participated in this stage. The proper grammar, appropriate and correct words and items’ scoring were assessed by experts.
  The quantitative content validity was evaluated using content validity ratio (CVR) and content validity index (CVI) [28]. For CVR calculation, 10 experts were invited to assess item essentiality. The score of each item was considered within a three-degree range of “not essential, useful but not essential, essential” from 1 to 3 points. The items with CVR values above 0.62 were retained in the instrument based on the Lawshe’s table [29]
  For the CVI calculation, the same experts were asked to evaluate the relevance and adequacy of the items based on 4-point Likert scale (not relevant, somewhat relevant, quite relevant, highly relevant). The Item-CVI (I-CVI) and Scale-CVI (S‐CVI) were calculated. I-CVI was computed by dividing the number of experts giving a rating score of either 3 or 4 by the total number of experts. Values of I-CVI more than 0.79 was considered acceptable and showed that the item is relevant [30]. The S-CVI acceptance criterion was between 0.8 and 0.9 [31].
ii. *Face validity* Qualitative and quantitative methods were used for face validity evaluation. In the qualitative approach, the items of the questionnaire were evaluated by ten infertile women regarding the difficulty level, proportion, clarity, and necessity of each item. For Quantitative face validity assessment, the impact score of each item was calculated (Impact Score = Frequency (%) × Importance). 10 infertile women scored the importance of each item with a 5-point scale from 1 (not important) to 5 (very important). The impact score of each item was calculated by multiplying its importance score by the number of participants who had rated it 4 or 5, and items with impact scores more than 1.5 were chosen for further analysis [28].
iii. *Pilot reliability* The initial internal consistency of the ISI-F was calculated in a pilot study. 50 women with primary infertility completed the questionnaire. Cronbach's alpha coefficient of each question and of the whole instrument were calculated. Items with an internal correlation value of less than 0.2 were removed. Following the pilot study, a 22 item pre-final version of the ISI-F was prepared for construct validity evaluation.
iv. *Construct validity* Exploratory factor analysis (EFA) was used to evaluate the construct validity and extract the latent constructs of ISI-F. Psychometric properties of the ISI-F were examined by conducting a cross-sectional study. 300 women who referred to the Isfahan Fertility and Infertility Center with known primary female infertility and without any psychological disorder completed the questionnaire. All the participants were informed about the study objectives and how to complete the questionnaire.

To identify the underlying components of the ISI-F items, Exploratory Factor Analysis (EFA) was conducted using the main methods of principal components analysis (PCA) and varimax rotation. The Kaiser-Meyer-Olkin (KMO) measure and Bartlett’s Test of Sphericity were used to determine the adequacy of the sample for factor analysis. To determine the number of potential underlying factors, eigenvalues greater than one and the scree plot were used. Factor loadings greater than or equal to 0.40 were considered appropriate [32]. Statistical calculations were performed using SPSS software (version 20, SPSS Inc., Chicago, USA).

### Reliability

Internal consistency and stability were used to verify the reliability of ISI-F. To evaluate the internal consistency of ISI-F, coefficients of Cronbach's alpha and McDonald's Omega were estimated, and values greater than 0.7 were considered acceptable [33, 34]. The test re-test method was used for assessment of stability. 30 infertile women completed the questionnaire during a two-week interval, and Intraclass Correlation Coefficient (ICC) was calculated. ICC values of 0.7–0.8 were considered as having suitable stability [32, 35].

## Results

Overall, 55 items of ISI-F were developed as a result of the qualitative study. In the second stage, considering CVR above 0.62 and I-CVI above 0.79, and as a result of the pilot study 22 items were remained in the questionnaire.

For exploratory factor analysis 300 infertile women completed the questionnaire. The age range was between 20 and 46 years old (31.34 ± 5.52). The mean of infertility duration was 5.27 ± 4.33 (range: 1–24 years). Demographic data are shown in Table 1. After expletory factor analysis two more items were removed, so the final questionnaire had 20 items. The S‐CVI and S-CVR score for all the items was calculated as 0.94 and 0.87 respectively. The details of CVI, CVR, and the items that remained in the questionnaire are presented in Table 2.

**Table1:** Demographic characteristics of the study participants (n = 300)

	Mean(SD^a^)/ N(%)
Age	31.43(5.52)
Educational level	
Elementary	26(8.6)
Diploma	119(39.7)
Academic	155(51.7)
Occupational status	
Employed	68(22.7)
Housewife	232(77.3)
Residency	
Urban	259(86.3)
Rural	41(13.7)
Infertility duration	5.27(4.33)
Infertility factor	
An ovulatory cycle & PCOD	176(58.7)
Fibromyoma	20(6.7)
Endometriosis	8(2.7)
Fallopian tube occlusion	18(6)
Uterine abnormality	2(0.7)
Unknown	44(14.6)
Other female factors	32(10.6)

**Table2:** Content analysis of the questionnaire including CVI, CVR and exploratory factor analysis of ISI-F

Factors and items of ISI-F	Factor loading			CVR^a^	CVI^b^
	F1	F2	F3		
*Factor1: stigma profile (7 items)* *Eigenvalue = 4.26, Variance = 21.34%*					
Others taunt me because of my infertility	.699^**c**^	.220	.204	0.87	0.92
Others’ feeling pity for my infertility is disturbing	**.801**	.138	.057	0.99	0.94
Others’ questions and curiosity about my infertility disturbs me	**.771**	.354	.091	0.86	0.98
I feel that others look at me differently because of my infertility	**.597**	.227	.345	0.89	0.94
Pressure from others and the society is more disturbing than infertility	**.703**	.245	.191	0.89	1.00
I do not like to be called “infertile”	.649	.242	.131	0.86	1.00
Other women, do not understand me	**.665**	.087	.175	0.81	0.80
*Factor2: self-stigma (6 items)* *Eigenvalue = 3.69, Variance = 18.47%*					
Infertility is considered as a defect for women	.076	.634	.169	0.82	1.00
I feel inferior to others because I cannot get pregnant	.228	.716	.205	0.84	0.98
I am worry that my husband would remarry or divorce me because I am infertile	.123	.534	.007	0.81	0.92
I want to have child as soon as possible to get rid of others’ negative words	.452	.551	.185	1.00	1.00
I avoid being in gatherings because of my infertility problem	.169	.680	.174	0.87	0.98
I feel sad when others have children	.051	.602	.228	0.84	0.80
*Factor3: escaping from stigma (7 items)* *Eigenvalue = 2.84, Variance = 14.19%*					
I make excuses for not getting pregnant (I have not decided to have children…)	.316	.076	.740	0.84	0.87
I do not want to talk to others about my infertility	.374	.133	.596	0.82	0.94
I hide my infertility problem from others	.146	.206	.822	0.86	1.00
I talk to my family about my infertility (R)^d^	.201	.168	.510	0.84	0.91
I hide my infertility problem from my in-laws	.023	.192	.721	0.90	0.98
I am worried that others know my infertility problem	.313	.161	.714	0.98	1.00
I try to keep my distance with others to avoid their interference	.350	.315	.518	1.00	0.99

^a^Content validity index

^b^Content validity ratio

^c^Bold numbers show the items’ loading of underlying fact

^d^Reverse scoring

**Fig. 2:**
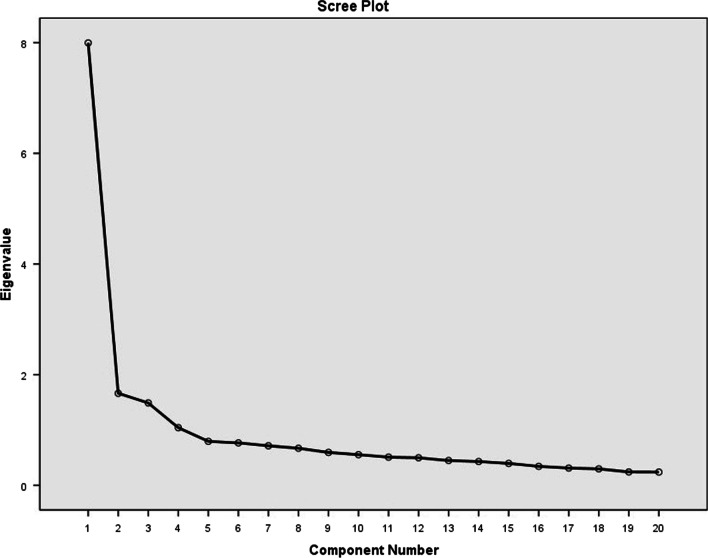
Scree-plot. Based on the Scree-plot, three factors were proposed for extraction in EFA for ISI-F

Based on principal component analysis (PCA), 22 items were refined in the EFA. The adequacy of sample size and data appropriateness were confirmed by Kaiser-Meyer-Olkin (KMO) and Bartlett’s Test of Sphericity (KMO = 0.911 and χ2 = 2619.034, *p* = 0.001). Three main factors were emerged with eigenvalues of greater than 1. Also the Scree-plot method confirmed the number of factors (Fig. 2). Minimum loading for the items to remain in the questionnaire was set to be 0.4. None of the correlations among the factors was greater than 0.60. Component loadings ranged from 0.45 to 0.80 (Table 2). Two items were removed because of low loading in factor analysis. Finally, 20 items were loaded in 3 main factors that explained 54.013% of the observed variance, representing factors were consisted of:

- Stigma profile (7 items)
- Self-stigma (6 items)
- Escaping from stigma (7 items). The results are shown in Table 2.

The Cronbach’s alpha and McDonald's Omega coefficient for the entire scale were 0.909 and 0.916 respectively. The results showed that all the factors had acceptable internal consistency. The stability of the ISI-F and its subscales as measured by the Intraclass Correlation Coefficient (ICC) was also found to be satisfactory (ICC = 0.878) (Table 3). The 20 items of the ISI-F were scored on a 5-point Likert scale. The scoring of item number 17 is reverse. Therefore, the total score of the scale can range from 20 to 100. A higher score indicates a greater perception of infertility stigmatization.

**Table 3:** Cronbach’s α coefficients and ICC for the ISI-F and its subscales

	Number of items	Cronbach’s α coefficients	McDonald's omega coefficients	Intraclass correlation coefficients (ICC), CI95%
Factor1: stigma profile	7	0.875	0.876	0.836 (0.642–0.875)
Factor2: self-stigma	6	0.757	0.794	0.915 (0.822–0.959)
Factor3: escaping from stigma	7	0.854	0.875	0.929 (0.851–0.966)
Total scale	20	0.909	0.916	0.878 (0.761–0.946)

## Discussion

An instrument with 20 items was developed for assessment of perceived female infertility stigma in order to evaluate this concept in three dimensions of “Stigma profile”, “Self-stigma” and “escaping from stigma”. Acceptable explained variance of the scale shows its ability to measure the concept of perceived infertility stigma among infertile women. Findings Confirmed that ISI-F is a valid instrument and had acceptable validity (content, face and construct) and reliability (internal consistency and stability).

The first factor of the instrument, Stigma profile, was consisted of 7 items. Items of this dimension was specified to the behavior of the society’s members toward an infertile woman and infertility, including verbal sarcasm, curiosities and inappropriate questions and type of look and approach that could impose mental pressure on infertile women. Studies have shown that this type of behavior exists in every society from outsiders in direct or indirect ways [36–39]. In this regard, Slade et al in their questionnaire have noticed different look and judgment and no specific infertility stigma have been mentioned [16]. In line with this structure, Fu et al extracted a dimension under the title of general stigma in their questionnaire in which curiosity and inappropriate questions have not been mentioned [13]; whilst this was one of the most important part of the present study. In the present study women have reported behaviors and approaches from other women. The item of “Other women, do not understand me” has been placed in the female infertility stigma instrument due to its emphasized role in imposing mental pressure on infertile women. Women had a bad feeling while being called “infertile” and did not like these nicknames. Most of them were reluctant to use the infertility term. The item of “I do not like to be called infertile” has been added regarding this feeling in women.

The second factor of the tool was self-stigma. It contained 6 items which was focused on the feelings, perceptions and attitude of women toward themselves in relation with infertility. Due to their infertility, these women feel isolated from the world of fertile women [5] in a way that inferiority complex, humiliation, isolation, social stigma, losing control and feeling flawed in association with infertility would become the center of the infertile women’s identity [4, 6]. Items of “infertility is considered as a defect for a woman” and “I feel inferior to others because I cannot get pregnant” are placed in this dimension. Infertility could have mental and social consequences for the individual and could affect the couple’s marital relationships [9, 19] The item of “I am worry that my husband would remarry or divorce me because I am infertile” is placed in this dimension. There are items in the Fu et al questionnaire about feeling shame and humiliation that are similar to the present questionnaire, but in the present questionnaire self-stigma has gone beyond these feelings and has considered aspects such as getting sad when others have children which is a sign of deep hidden feeling of humiliation; this could be considered as the strength of this instrument.

The third factor of the instrument was escaping from stigma. This factor contained 7 items which was about the defense mechanisms and family support, such as “I make excuses for not getting pregnant (I have not decided to have children…)”, “I talk to my family about my infertility”. In this factor common defense mechanisms of infertile women including secrecy, keeping distance with others and making excuses are mentioned. In the questionnaire by Slade et al this subject has not been mentioned at all [16]. In the questionnaire by Fu et al keeping distance with others and secrecy have been considered [13].

Regarding family’s support, women’s willingness to talk to their families was one of the items that remained in the ISI-F. There are items about women family in the Fu et al questionnaire considered as the stigma pressure. For example, “I feel like a burden to my family”, which has been mentioned the Fu et al questionnaire, while was not mentioned by any of the interviewed women in the present research, on the contrary, women had mostly consulted with their families which was mentioned as “I talk to my family about my infertility” in the Female Infertility Stigma Instrument (ISI-F).

It seems that the designed questionnaire, while having fewer items (20 items), which is considered as an important advantage, it is able to cover more aspects of infertility stigma and is more consistent with infertile women’s experiences.

The designed instrument had a high content validity which indicates that it has been able to evaluate the concept of female infertility stigma successfully. Also, this instrument had a significant reliability and stability which are considered as the strengths of this tool. As far as our study show, this instrument is the first tool that has been designed to evaluate female infertility stigma which has been designed using a mixed method study. Therefore, it could be a ground for further investigations in this field. Studied women were selected from one center which is the country’s referral center and people would visit it from different parts of the country, therefore it could be a representative of women’s experiences from different cultures and social backgrounds.

## Limitations

This study evaluated the perceived stigma and it is possible that, due to the sensitivity of the subject, feelings such as sympathy have also been perceived as stigma and therefore the real level of stigma might be lower than what they have perceived; this subject requires further investigations. Also, it is recommended that male stigma would be investigated and a Specific tool would be designed for this purpose too.

## Conclusions

A 20 items instrument was developed in this mix method study based on theoretical knowledge are more likely to be effective in the evaluating perceived female infertility stigma. The Infertility Stigma Instrument for Female (ISI-F) had appropriate coherence and desirable reliability and validity.

## Data Availability

The datasets used and/or analyzed during the current study available from the corresponding author on reasonable request.

## Abbreviations

ISI-F: Female infertility stigma instrument
MMARS: The mixed methods article reporting standards
CVR: Content validity ratio
CVI: Content validity index
EFA: Exploratory factor analysis
PCA: Principal components analysis
KMO: Kaiser-Meyer-Olkin
ICC: Intraclass correlation coefficient
SPSS: Statistical package for social science
